# A literature review of the impact of exclusion criteria on generalizability of clinical trial findings to patients with chronic pain

**DOI:** 10.1097/PR9.0000000000001050

**Published:** 2022-11-11

**Authors:** Vafi Salmasi, Theresa R. Lii, Keith Humphreys, Vinay Reddy, Sean C. Mackey

**Affiliations:** Departments of a Anesthesiology, Perioperative and Pain Medicine and; b Psychiatry and Behavioral Sciences, Stanford University School of Medicine, Palo Alto, CA, USA; c Center for Innovation to Implementation, Palo Alto Veterans Affairs Healthcare System, Palo Alto, CA, USA

**Keywords:** Eligibility criteria, Exclusion criteria, Generalizability, Pain, Psychological

## Abstract

The ability of clinical trials to inform the care of chronic pain may be limited if only an unrepresentative subset of patients are allowed to enroll. We summarize and report new insights on published studies that report on how trial exclusions affect the generalizability of their results. We conducted a PubMed search on the following terms: ((“eligibility criteria” AND generalizability) OR (“exclusion criteria” AND generalizability) OR “exclusion criteria”[ti] OR “eligibility criteria”[ti]) AND pain. We only considered studies relevant if they analyzed data on (1) the prevalence and nature of exclusion criteria or (2) the impact of exclusion criteria on sample representativeness or study results. The 4 articles that were identified reported differences in patients who were included and excluded in different clinical trials: excluded patients were older, less likely to have a paid job, had more functional limitations at baseline, and used strong opioids more often. The clinical significance of these differences remains unclear. The pain medicine literature has very few published studies on the prevalence and impact of exclusion criteria, and the outcomes of excluded patients are rarely tracked. The frequent use of psychosocial exclusions is especially compromising to generalizability because chronic pain commonly co-occurs with psychiatric comorbidities. Inclusion of more representative patients in research samples can reduce recruitment barriers and broaden the generalizability of findings in patients with chronic pain. We also call for more studies that examine the use of exclusion criteria in chronic pain trials to better understand their implications.

## Abstract 2

Patients with chronic pain are frequently excluded from trials because of comorbidities. More inclusive studies will have better generalizability considering differences between excluded and included patients.

## 1. Introduction

Roughly 20% of adults in the United States suffer from chronic pain, with 8% experiencing high-impact chronic pain^3^; this costs the economy more than $600 billion annually.^20^ Patients with chronic pain report more medical comorbidities than patients without chronic pain.^4,28^ Chronic pain also co-occurs frequently with psychiatric disorders such as depression and anxiety.^2,14,26^ Yet, randomized controlled trials (RCTs) for chronic pain disorders often exclude patients with comorbidities in the pursuit of participant safety, reducing treatment response heterogeneity, and increasing statistical efficiency. This exclusionary approach, while often necessary for efficacy or mechanistic studies, comes at the cost of having study samples that poorly represent the real-world population with the studied disease. The nature of the research question usually determines if the eligibility criteria need to be more exclusive vs more inclusive. Explanatory trials determine efficacy of an intervention under ideal circumstances and thus requires a more homogeneous population. However, these trials have to be followed by more pragmatic trials to determine the effectiveness of the same intervention under general conditions that it is intended to be applied.^22,24^

The degree to which the results from a study can be applied to a larger or more heterogeneous population is referred to as generalizability, or external validity. In recent years, the generalizability of RCTs has come under scrutiny in several disciplines, including psychiatry,^9,10,29^ rheumatology,^15,27^ and infectious disease.^6,21^ Whether this same problem occurs in pain medicine has not been studied. We therefore reviewed available studies that evaluate differences in baseline characteristics or outcomes between included and excluded participants in chronic pain trials, and we summarize the impact of these exclusions on study results when applicable.

## 2. Methods

We closely emulated the methods of the Cross-disease Review of Exclusion Across Medicine (CREAM) project previously presented by Humphreys and colleagues. Their project assessed the impact of exclusion criteria in research conducted across a range of medical specialties. A detailed description of the literature review procedure can be found in the article on schizophrenia trials by Humphreys.^10^ We applied the same search strategy and terms used by the CREAM project, but with a different condition of interest (pain). We did not preregister our protocol because our methods more closely resemble a literature review, rather than a systematic review.

**Figure 1.**
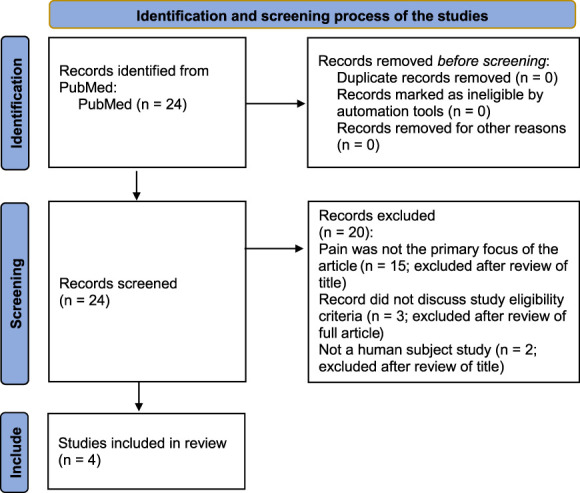
Preferred Reporting Items for Systematic Reviews and Meta-Analyses (PRISMA) flow diagram.

Literature was primarily identified by conducting English-language searches in PubMed (original date of search: November 14, 2020) on the following terms: “Eligibility criteria and generalizability” (anywhere in article), “exclusion criteria and generalizability” (anywhere in article), “exclusion criteria” (in the title of article) and “eligibility criteria” (in the title of article) (ie. ((“eligibility criteria” AND generalizability) OR (“exclusion criteria” AND generalizability) OR “exclusion criteria”[ti] OR “eligibility criteria”[ti]) AND pain). This generated 822 articles. Like other CREAM projects, we then focused on patients with chronic pain by adding the search term “pain (anywhere in the article)” to the other search terms; this resulted in 24 articles. We considered studies relevant if they analyzed data on (1) the prevalence and nature of exclusion criteria and/or (2) the impact of exclusion criteria on sample representativeness or study results. We excluded studies that only reported their exclusion criteria and rate. The first authors (V.S. and T.L.) independently reviewed all these articles and their references to other studies of exclusion criteria (Fig. 1).^17^ Both authors chose similar studies that met our relevance criteria.

## 3. Results

One clinical trial and 3 review articles (total N = 4) met our relevance criteria.^1,11,13,18^ The clinical trial evaluated differences in baseline characteristics and outcomes between included and excluded participants. The remaining articles reported on the prevalence and nature of exclusion criteria. Disease entities covered include low back pain (2 articles),^1,13^ knee osteoarthritis (1 article),^11^ and postoperative pain after hip and knee arthroplasty (1 article).^18^

Maas et al. analyzed the outcomes of 1148 participants in a pragmatic multicenter trial to treat low back pain. They compared the outcomes of a randomized group (n = 241) to 2 observational groups who received the studied treatment without randomization. Observational group 1 (n = 455) consisted of participants who fulfilled all eligibility criteria but were not randomized. Observational group 2 (n = 452) consisted of participants who did not meet eligibility criteria. Both observational groups 1 and 2 received the study treatment. Their sample was drawn from 16 Dutch clinics and 102 physiotherapy practices. The authors explain that the eligibility criteria of the randomized group were “standardized” and they were similar to the ones used by pain physicians in clinical practice in the Netherlands.^13^

The most frequently applied exclusion was “psychological problems” which prevented 60.8% of participants in observational group 2 from enrolling in the RCT, including those with other exclusions (17.5%). Age and body mass index were the second (31.2%) and third (16.2%) most frequently applied exclusions, respectively. Participants with multiple overlapping criteria made up 19.2% of excluded participants. The combined effect of all criteria was to exclude 452 of 1148 (39.4%) participants who had otherwise been deemed eligible during initial screenings but were instead diverted to observational group 2. Compared to participants in the randomized group and observational group 1, those in observational group 2 were older, less likely to have a paid job, had more functional limitations at baseline, and used strong opioids more often.^13^

Regarding outcomes from the study by Maas et al., patients who had pain from facet joints had slightly less pain relief if they were in observational group 2 compared with randomized patients (mean difference of 0.4 on a numeric scale of 1–10). No differences in pain scores were found in observational group 2 patients who had pain from sacroiliac (SI) joints. Patients in observational group 2 also had slightly less improvement in functional status (mean difference of 5.83 and 7.16 on the Oswestry Disability Index [pain from facet joints and SI joints, respectively]), and a lesser likelihood of perceiving treatment success (odds ratio 0.67 and 0.72 [pain from facet joints and SI joints, respectively]). The authors report that these statistically significant differences were too small to be clinically relevant.^13^

Koog et al. reviewed 355 RCTs for knee osteoarthritis which were published up to December 2011. They divided exclusion criteria into “non–osteoarthritis-related” and “osteoarthritis-related” reasons. Ten exclusion criteria were identified in the non–osteoarthritis-related category. Inability to give informed consent was the most commonly applied non–osteoarthritis-related exclusion (used in 79.4% of trials), followed by recent exposure to other potential treatments (61.4%) and medical conditions that make it difficult to participate in a trial (57.2%) or might pose a risk to receiving treatment (56.0%). Other psychological or psychiatric exclusions were not identified in the review; however, it is unclear whether these were included under “medical conditions.” In the osteoarthritis-related category, 9 overlapping exclusion criteria were identified. Lower boundaries of age were the most common osteoarthritis-related exclusion (58.9%), followed by exclusion of nonpainful osteoarthritis (58.3%) and limits on osteoarthritis severity (47.3%). Koog et al.^11^ did not tabulate the number of included or excluded participants, nor did they compare baseline characteristics or outcomes of excluded participants to included participants.

Amundsen et al. reviewed 168 RCTs for chronic low back pain published between 2006 and 2012. Exclusion criteria were noted to be highly diverse, so the authors categorized exclusion criteria into 19 themes. The most ubiquitous exclusion themes were causes of back pain from other conditions, including malignancy (46.4% of trials) or trauma (37.5%). Pregnancy was a common exclusion criterion (48.2%) as was having previous or scheduled surgery (50%). Noteworthy exclusion criteria frequently encountered in real-world patients include psychosocial conditions (ie, psychiatric disorders, depression, severe psychiatric disorder, and impaired cognition) (34.5%) and medicolegal issues (23.2%). Amundsen et al. did not tabulate the number of included or excluded participants, nor did they compare baseline characteristics or outcomes of excluded participants to included participants.^1^

Finally, Pedersen et al. analyzed the exclusion criteria used in 550 RCTs investigating postoperative pain treatment for hip and knee total arthroplasty. The authors also compared baseline characteristics from 48,962 research participants enrolled in RCTs with an unrandomized clinical cohort from the Danish Hip and Knee Arthroplasty Registries from 2005 to 2019. The authors found that the most commonly used exclusion criteria were having a high American Society of Anesthesiologists (ASA) Physical Status Classification score (62% of trials), older age (45%), chronic opioid use (41%), neurological disorders (41%), and renal disease (40%). Psychiatric or cognitive exclusions were used in 29% of trials.^18^

Research participants were on average younger than their clinical counterparts; however, body mass index (BMI) American Society of Anesthesiologists (ASA) physical status scores, and sex distribution were comparable. When examining trends over time, the enrolled research participants' average age has increased, and in the subgroup of patients undergoing total knee arthroplasty, BMI and ASA scores have also increased. Meanwhile, age, BMI, and ASA scores in the research cohort have not changed, although fewer women are being included in total hip arthroplasty trials over time. The prevalence of other exclusion criteria (such as chronic opioid use or medical and psychiatric comorbidities) was not reported in either cohort. The total number of included or excluded participants from the research cohort was not reported either.^18^

## 4. Discussion

The current review found very few published studies on the prevalence and impact of exclusion criteria in clinical trials for chronic pain. Disease entities were limited to knee osteoarthritis, pain after hip and knee arthroplasty, and chronic low back pain.^1,11,13,18^ This leaves out large swaths of other common chronic pain conditions such as migraine, lumbar radiculopathy, and peripheral neuropathy. One study compared the outcomes of randomized patients with excluded patients who had received the studied treatment outside of the RCT.^13^ Three review articles reported on the nature and prevalence of exclusion criteria used in clinical trials^1,11,18^; only one of them analyzed differences in baseline characteristics by comparing trial patients with a national database of nontrial patients.^18^ None of the review articles estimated exclusion rates, probably because the number of patients excluded before randomization is inconsistently reported by RCTs.^1,11,18^

The authors of the study that followed excluded patients with chronic low back pain argued that the differences in outcomes between randomized and excluded patients were too small to be clinically relevant, despite reaching statistical significance. We agree that the mean differences seen with numeric pain scores and Oswestry Disability Index scores are relatively inconsequential.^13^ Exclusions are usually designed assuming that they result in more heterogeneous outcomes. The interpretation of the authors shows that this assumption is wrong and more strict exclusion criteria were unnecessary. However, we believe that the difference in perceived treatment success is clinically impactful because patients who believe that their treatment was unsuccessful are more likely to seek additional medical care and less likely to return to work, with resultant costs to the healthcare system and society.

Another way to interpret the difference in perceived treatment success is by calculating the number needed to harm (NNH) for having exclusionary comorbidities. Number needed to harm measures how many people need to be exposed to a risk factor for 1 person to experience an adverse effect or harm. A lower NNH indicates a greater risk of harm. In this case, if we define “harm” as a lack of perceived treatment success 3 months after receiving treatment, we can use the formula NNH = 1/(I_E_ − I_0_), where I_E_ is the incidence of harm in those with exclusionary comorbidities and I_0_ is the incidence of harm in those without exclusionary comorbidities. Based on data reported by Maas et al., the NNH for having exclusionary comorbidities was 8 for patients with pain from facet joints and 12 for patients with pain from SI joints. This means that for every 8 patients with exclusionary comorbidities receiving treatment for facet joint pain, 1 patient will report a lack of treatment success 3 months after treatment. Likewise, for every 12 patients with exclusionary comorbidities receiving treatment for SI joint pain, 1 patient will report a lack of treatment success 3 months after treatment. As a point of reference, the NNH for gabapentin causing somnolence^7^ is reported to be 11.

One class of exclusions that stood out in our review was psychiatric and psychosocial problems. Approximately one-third of all low back pain trials excluded participants with psychological issues. This proportion was slightly less in trials for pain after hip and knee arthroplasty. Chronic pain is frequently comorbid with psychiatric disorders such as depression and anxiety, and the presence of adverse psychosocial factors portends a worse prognosis in both surgical^8,23,25^ and nonsurgical^5,12,16^ populations. By excluding these patients, the researchers hope to reduce heterogeneity in treatment response; however, this practice likely results in overestimating treatment efficacy in a general chronic pain population. Some clinicians may reserve procedural interventions for patients without psychiatric disease, making their clinical population more comparable to research populations. However, this still leaves a broad category of medication-based and behavioral-based treatments, for which the exclusion criteria used in RCTs is not well characterized.

In addition, many studies did not report the details of psychiatric comorbidities and potentially combined disparate diagnoses into 1 group. We observe a similar pattern in the vague descriptions of “medical comorbidities” too.^11^ We believe that it is essential for clinicians to know the nature and details of “medical and psychiatric” comorbidities when generalizing the result of a clinical trial to patient they are treating. It is for example important to know if a clinical trial excluded all patient with depressive symptoms or only patients with major depressive disorder; this knowledge can have a significant impact in the decision-making process of clinicians when generalizing the data.

## 5. Conclusion

Although the research question and type of clinical trial (eg, explanatory vs pragmatic) can influence the choice of eligibility criteria, overly restrictive exclusion criteria and exclusion of highly prevalent comorbidities may also threaten the external validity of randomized controlled trials. In the field of pain management, there are very few published studies on the prevalence and impact of exclusion criteria, and the outcomes of patients excluded from randomization are rarely tracked. The frequent use of psychiatric and psychosocial exclusions in chronic pain trials is especially concerning because chronic pain commonly co-occurs with—and in some cases, perpetuates these issues. We call for more studies that examine the use of exclusion criteria in chronic pain trials. Understanding the implications of exclusions in these trials is particularly pertinent given the complexities of patients with chronic pain. Ultimately, such studies are important to determine whether the inclusion of more representative patients in research samples can reduce recruitment barriers and broaden the generalizability of study findings across chronic pain populations.

We also recommend more transparent reporting of exclusion criteria in chronic pain trials because this will promote interpretability of results and facilitate future research on the effects of exclusion criteria. Finally, we call for more inclusive studies with less stringent exclusion criteria because this will promote generalizability of results to real-world patient populations. Although increased heterogeneity in the study population will necessitate larger sample sizes to discern an effect of an intervention, pragmatic trial designs embedded into existing practice settings can be used to recruit large numbers of participants at lower cost than conventional trials.^19^

## Disclosures

The authors have no conflict of interest to declare.
